# Accessory hepatic vein recanalization for Budd-Chiari syndrome: a systematic review and meta-analysis

**DOI:** 10.1186/s12876-023-02969-z

**Published:** 2023-10-02

**Authors:** Lu-Lu Lv, Han-Bo Xu, Sheng-Li Li, Peng Xu

**Affiliations:** 1 Department of Radiology, Xuzhou Central Hospital, Xuzhou, China; 2 Department of Radiology, Affiliated Hospital of Xuzhou Medical University, Xuzhou, China; 3 Clinical Research Institute, Affiliated Hospital of Xuzhou Medical University, Xuzhou, China

**Keywords:** Budd-Chiari syndrome, Accessory hepatic vein, Recanalization, Meta-analysis

## Abstract

**Background:**

Budd-Chiari syndrome (BCS) results when the outflow of the hepatic vein (HV) is obstructed. BCS patients exhibiting an accessory HV (AHV) that is dilated but obstructed can achieve significant alleviation of liver congestion after undergoing AHV recanalization. This meta-analysis was developed to explore the clinical efficacy of AHV recanalization in patients with BCS.

**Materials and methods:**

PubMed, Embase, and Wanfang databases were searched for relevant studies published as of November 2022, and RevMan 5.3 and Stata 12.0 were used for pooled endpoint analyses.

**Results:**

Twelve total studies were identified for analysis. Pooled primary clinical success, re-stenosis, 1- and 5-year primary patency, 1- and 5-year secondary patency, 1-year overall survival (OS), and 5-year OS rates of patients in these studies following AHV recanalization were 96%, 17%, 91%, 75%, 98%, 91%, 97%, and 96%, respectively. Patients also exhibited a significant reduction in AHV pressure after recanalization relative to preoperative levels (*P* < 0.00001). Endpoints exhibiting significant heterogeneity among these studies included, AHV pressure (I^2^ = 95%), 1-year primary patency (I^2^ = 51.2%), and 5-year primary patency (I^2^ = 62.4%). Relative to HV recanalization, AHV recanalization was related to a lower rate of re-stenosis (*P* = 0.002) and longer primary patency (*P* < 0.00001), but was not associated with any improvements in clinical success (*P* = 0.88) or OS (*P* = 0.29) relative to HV recanalization.

**Conclusions:**

The present meta-analysis highlights AHV recanalization as an effective means of achieving positive long-term outcomes in patients affected by BCS, potentially achieving better long-term results than those associated with HV recanalization.

**Supplementary Information:**

The online version contains supplementary material available at 10.1186/s12876-023-02969-z.

## Background

Budd-Chiari syndrome (BCS) results when the outflow of the hepatic vein (HV) is obstructed [1–3]. Recanalization of one HV can effectively alleviate the liver congestion experienced by BCS patients in whom three HVs are obstructed [4–6]. When all three of these HVs exhibit diffuse or long-segmental obstruction, however, it is not possible to perform such HV recanalization. In these cases, transjugular intrahepatic portosystemic shunt (TIPS) insertion is generally employed as an alternative therapeutic intervention [7–9].

Accessory HV (AHV) dilation has been identified as a key compensatory mechanism that is engaged in some patients with BCS [10]. When patients exhibit an AHV that is dilated but obstructed, liver congestion can be effectively alleviated via AHV recanalization [11–22]. Even so, the long-term outcomes that these BCS patients experience following AHV recanalization remain incompletely understood. Accordingly, a systematic meta-analysis is warranted to clarify the short- and long-term efficacy of AHV recanalization as a therapeutic intervention aimed at alleviating the symptoms of BCS.

This meta-analysis was designed to explore the clinical efficacy of AHV recanalization as a treatment for BCS patients.

## Methods

### Study design

The Observational Studies in Epidemiology (MOOSE) checklists were used to guide this meta-analysis [23], which was registered at INPLASY.COM (No. INPLASY2022110071).

PubMed, Embase, and Wanfang databases were searched for relevant studies published by November 2022 with the search strategy: (((Budd Chiari syndrome[Title/Abstract]) OR (BCS[Title/Abstract])) AND ((accessory hepatic vein[Title/Abstract]) OR (AHV[Title/Abstract]))) AND (recanalization[Title/Abstract]).

Studies eligible for inclusion were those that (i) reported data pertaining to AHV recanalization in BCS patients and (ii) reported on at least one outcome of interest including clinical success rates, pre- and postoperative AHV pressure, primary and/or secondary patency rates, restenosis rates, and overall survival (OS) rates. Studies were excluded if they included < 10 patients or were meta-analyses, reviews, or case reports. No language restrictions were imposed on study inclusion. Meeting abstracts can be included if they fulfill the inclusion criteria.

### Data extraction

Two investigators independently extracted data from included studies, with a third investigator resolving any discrepancies. Extracted data included study details (first author, year, study design, quality assessment), baseline patient data (number of patients, age, sex, symptoms, AHV diameter, Child–Pugh scores, treatment methods, follow-up interval), and outcome data (AHV pressure, clinical success rates, restenosis rates, and 1- and 5-year primary patency, secondary patency, and OS rates). The restenosis rate was the primary endpoint for this study.

### Quality analyses

The Newcastle–Ottawa scale was used to determine the quality of all retrospective analyses [24]. Briefly, studies were scored based on criteria pertaining to selection, comparability, and outcomes (4, 2, and 3 points each), with high-quality studies being those with a final score ≥ 7.

### Definitions

Clinical success of AHV recanalization is defined as if patients experienced the alleviation of BCS symptoms and improved hepatic function within 7 days following recanalization [19, 22]. Rates of primary patency were evaluated as the interval between the time of recanalization and the time of re-obstruction [22]. Secondary patency was defined by the period between recanalization and the second instance of re-obstruction [22]. OS was defined as the interval between recanalization and all-cause death [22].

### Statistical analyses

RevMan 5.3 and Stata 12.0 were used to pool comparative data and individual rates, respectively. The Q test and the I^2^ statistic were used when evaluating heterogeneity, and significant heterogeneity was defined by an I^2^ value > 50%. Sensitivity analyses were performed via a “leave-one-out” approach to identify drivers of heterogeneity. Publication bias was examined using Egger’s test if the included studies ≥ 10. Otherwise, the funnel plot was used to assess the publication bias. *P* < 0.05 was the significance threshold.

## Results

### Study selection

**Fig. 1:**
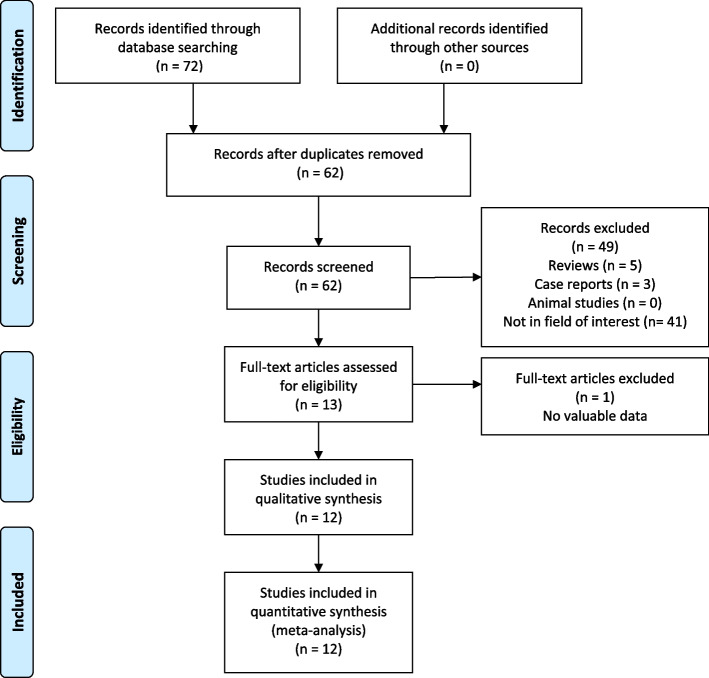
The flow chart of this meta-analysis

A flowchart for this meta-analysis is provided in Fig. 1. Initial searching identified 72 studies, of which 12 were incorporated into the final meta-analysis (Table 1). All 12 studies were performed in China and exhibited Newcastle–Ottawa Scale values ranging from 6–8.

**Table 1:** Baseline data of the included studies

First author	Publication year	Countries	Study design	Newcastle–Ottawa Scale
1/Chen [11]	2018	China	Retrospective	8
2/Cui [12]	2016	China	Retrospective	6
3/Fu [13]	2014	China	Retrospective	6
4/Fu Y [14]	2015	China	Retrospective	7
5/Li D [15]	2021	China	Retrospective	7
6/Li H [16]	2021	China	Retrospective	7
7/Li J [17]	2018	China	Retrospective	7
8/Liu [18]	2021	China	Retrospective	7
9/Lv [19]	2021	China	Retrospective	8
10/Qi [20]	2017	China	Retrospective	7
11/Wang [21]	2015	China	Retrospective	7
12/Xia [22]	2022	China	Retrospective	8

In total, 377 patients with BCS underwent AHV recanalization across these 12 studies (Table 2). Mean AHV diameter values ranged from 7.7 – 11.3 mm. AHV balloon dilation was performed in 302 patients, while 75 underwent stent insertion in the AHV. All patients were treated with low molecular weight heparin with followed oral warfarin after treatment.

**Table 2:** Baseline data of the patients in the included studies

First author	Patients number	M/F	Age (y)	AHV diameter (mm)	Nature of obstruction		Symptoms	Co-morbidity	Child–Pugh score	Operators	Treatments		Follow-up (months)
					MO	SO					Balloon	Stent	
1/Chen [11]	18	11/7	34.7	NG	18	0	AD, AP, A,GB	NG	8.1	IR	16	2	29.4
2/Cui [12]	28	NG	NG	NG	28	0	AD, AP, A,GB	LC: 18	8.1	IR	23	5	33.9
3/Fu [13]	14	NG	36.9	9.9	NG	NG	AD, AP, A,GB	NG	NG	IR	12	2	13.7
4/Fu Y [14]	20	11/9	33.4	8.5	20	0	AD, AP, A,GB	NG	NG	IR	18	2	15.8
5/Li D [15]	46	27/19	36.1	8.1	NG	NG	AD, AP, A,GB	NG	8	IR	40	6	30.3
6/Li H [16]	68	35/33	39.2	8	NG	NG	AD, AP, A,GB	AS: 4; H: 3	8	IR	52	16	60.2
7/Li J [17]	60	36/24	39.5	11.3	52	8	AD, AP, A,GB	LLE: 4	NG	VS	51	9	37
8/Liu [18]	21	9/12	40.3	9	21	0	AD, AP, A,GB	GV: 5; LLE: 8	NG	VS	21	0	27
9/Lv [19]	25	14/11	31.4	7.7	23	2	AD, AP, A,GB	NG	8.1	IR	20	5	NG
10/Qi [20]	20	11/9	36.9	10.2	20	0	AD, AP, A,GB	LLE: 1; H: 3	NG	IR	0	20	32.1
11/Wang [21]	30	16/14	34.3	8.4	30	0	AD, AP, A,GB	NG	NG	IR	27	3	34.2
12/Xia [22]	27	15/12	32.4	8	25	2	AD, AP, A,GB	NG	8.1	IR	22	5	NG

*M* male, *F* female, *AHV* accessory hepatic vein, *MO* membranous obstruction, *SO* segmental obstruction, *AD* abdominal distention, *AP* abdominal pain, *A* ascites, *GB* gastrointestinal bleeding, *LC* liver cirrhosis, *AS* antiphospholipid syndrome, *H* hyperhomocysteinemia, *GV* gastric varix, *LLE* lower limb edema, *IR* interventional radiologist, *VS* vascular surgeon, *NG* not given

### Clinical success rates

**Fig. 2:**
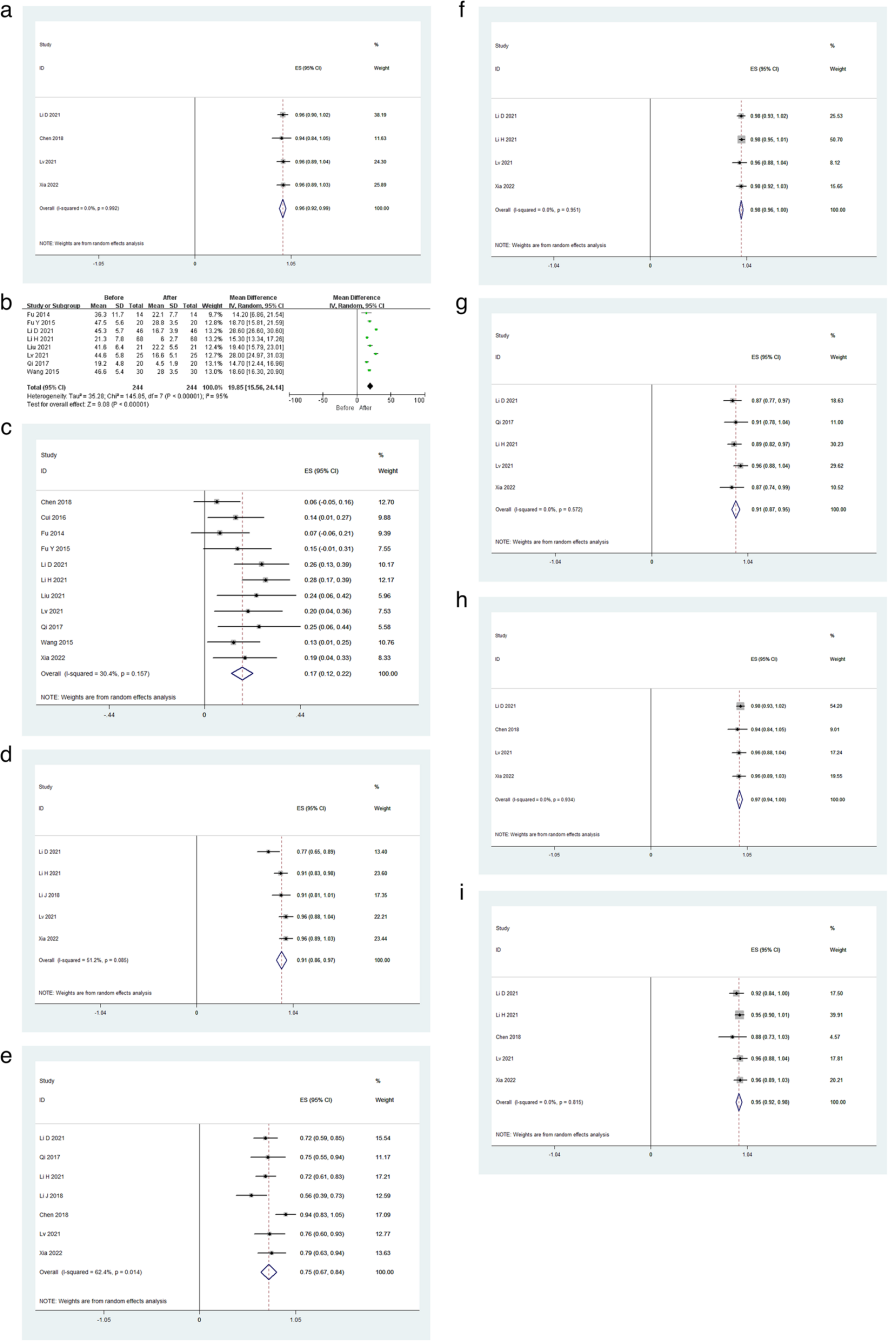
Pooled results for (**a**) clinical success rate, **b** AHV pressure before and after recanalization, **c** re-stenosis rate, **d** 1-year primary patency rate, **e** 5-year primary patency rate, **f** 1-year secondary patency rate, **g** 5-year secondary patency rate, **h** 1-year OS rate, and **i** 5-year OS rate

Based on the results of four studies [11, 15, 19, 22], the pooled clinical success rate of AHV recanalization was 96% (95% CI: 92%-99%, Fig. 2a). This endpoint was not subject to significant heterogeneity (I^2^ = 0.0%), but publication bias was significant on the funnel plot (supplement Fig. 1a). We found the source of publocation bias is Lv et al. [19] study.

### AHV pressure

Based on findings from seven studies [13–16, 18–21], pooled AHV pressure decreased significantly after recanalization relative to pre-procedure levels (MD: 19.85; 95% CI: 15.56–24.14, *P* < 0.00001, Fig. 2b). While this endpoint was subject to significant heterogeneity (I^2^ = 95%), the source of such heterogeneity was not identified through sensitivity analysis, and results were no subject to publication bias ( supplement Fig. 1b).

### Restenosis rates

Based on the results of 11 studies [11–16, 18–22], the pooled AHV re-stenosis rate was 17% (95% CI: 12%-22%, Fig. 2c). This endpoint was not subject to significant heterogeneity (I^2^ = 30.4%), nor was there publication bias (*P* = 0.066).

### Primary patency rates

Based on the results of five studies [15–17, 19, 22], the pooled 1-year AHV primary patency rate was 91% (95% CI: 86%-97%, Fig. 2d). This endpoint was subject to significant heterogeneity (I^2^ = 51.2%), which sensitivity analyses indicated was derived from the study conducted by Li et al. [15]. These findings were also subject to significant publication bias ( supplement Fig. 1c). However, the source of publication bias was not identified.

Based on the results of seven studies [11, 15–17, 19, 20, 22], the pooled 5-year AHV primary patency rate was 75% (95% CI: 67%-84%, Fig. 2e). This endpoint was subject to significant heterogeneity (I^2^ = 62.4%), which sensitivity analyses indicated was derived from the study conducted by Chen et al. [11]. These findings were also subject to significant publication bias (supplement Fig. 1d). However, the source of publication bias was not identified.

### Secondary patency rates

Based on the results of four studies [15, 16, 19, 22], the pooled 1-year secondary patency rate was 98% (95% CI: 96%-100%, Fig. 2f). This endpoint was not subject to significant heterogeneity (I^2^ = 0%), but publication bias was significant (supplement Fig. 1e). We found the source of publocation bias is Li et al. [15] study.

Based on the results of five studies [15, 16, 19, 20, 22], the pooled 5-year secondary patency rate was 91% (95% CI: 87%-95%, Fig. 2g). This endpoint was not subject to significant heterogeneity (I^2^ = 0%), but publication bias was significant (supplement Fig. 1f). However, the source of publication bias was not identified.

### OS rates

Based on the results of four studies [11, 15, 19, 22], the pooled 1-year OS rate was 97% (95% CI: 94%-100%, Fig. 2h). This endpoint was not subject to significant heterogeneity (I^2^ = 0.0%), but publication bias was significant (supplement Fig. 1g). We found the source of publocation bias is Lv et al. [19] study.

Based on the results of five studies [11, 15, 16, 19, 22], the pooled 5-year OS rate was 95% (95% CI: 92%-98%, Fig. 2i). This endpoint was not subject to significant heterogeneity (I^2^ = 0.0%), but there was significant publication bias (supplement Fig. 1h). However, the source of publication bias was not identified.

### Comparisons of AHV and HV recanalization

**Fig. 3:**
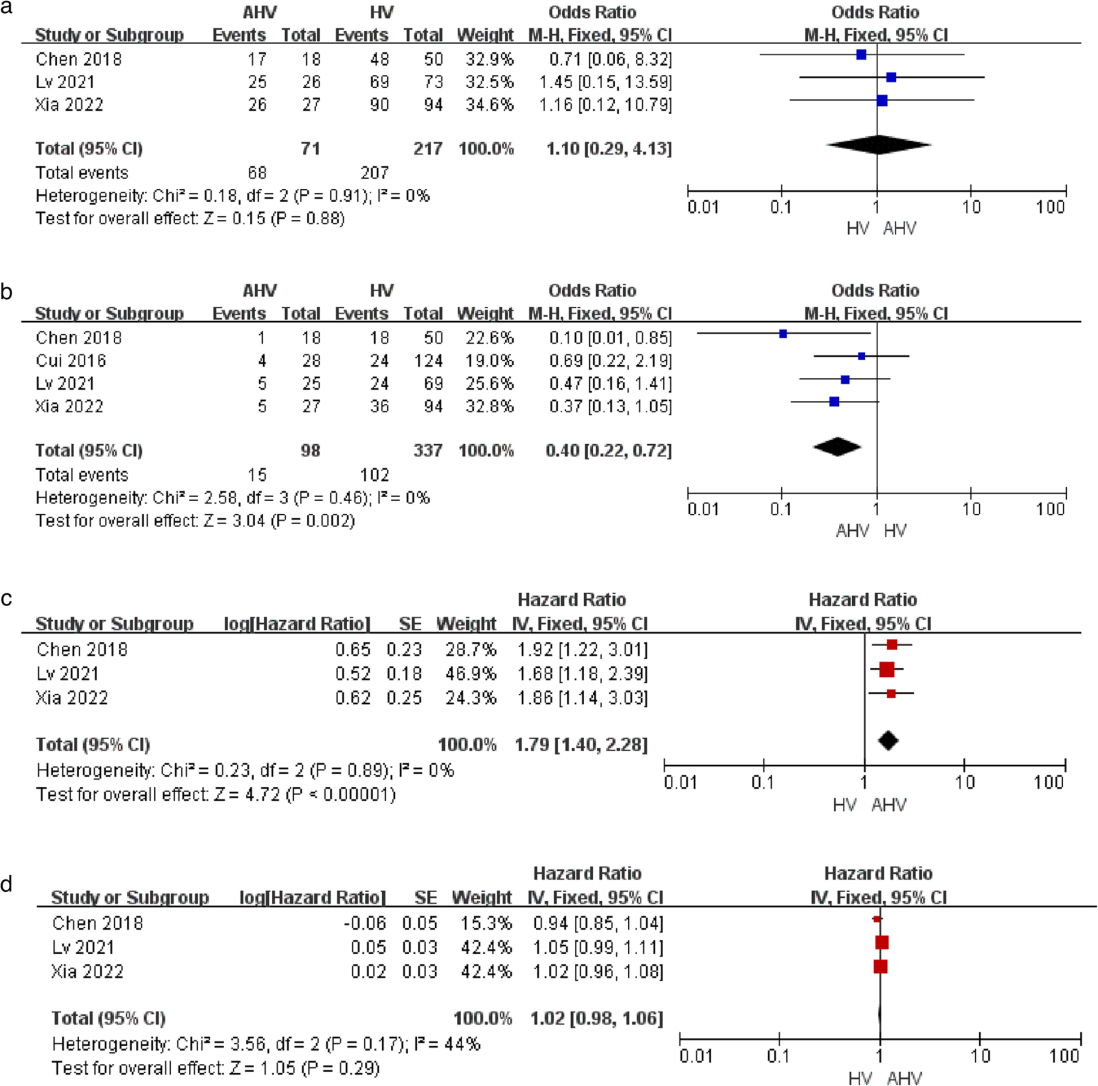
Pooled results for (**a**) clinical success rates, **b** re-stenosis rates, **c** primary patency duration, and **d** OS duration between AHV and HV groups

Clinical success rates were compared between AHV and HV recanalization procedures in BCS patients in three studies [11, 19, 22], revealing comparable pooled success rates in both groups (OR: 1.10; 95% CI: 0.29–4.13, *P* = 0.88, Fig. 3a). This endpoint was not subject to significant heterogeneity (I^2^ = 0%), nor was there publication bias (supplement Fig. 1i).

Restenosis rates were compared between AHV and HV recanalization procedures in four studies [11, 12, 19, 22], revealing a lower pooled restenosis rate in the AHV group relative to the HV group (OR: 0.40; 95% CI: 0.22–0.72, *P* = 0.002, Fig. 3b). This endpoint was not subject to significant heterogeneity (I^2^ = 0%), nor was there publication bias (supplement Fig. 1j).

It was possible to extract primary patency logHR values from three studies [11, 19, 22], revealing significantly longer primary patency in the AHV group relative to the HV group (HR: 1.79; 95% CI: 1.40–2.28, *P* < 0.000001, Fig. 3c). This endpoint was not subject to significant heterogeneity (I^2^ = 0%), nor was there publication bias (supplement Fig. 1k).

It was possible to extract OS logHR values from three studies [11, 19, 22], revealing no difference in OS rates between the AHV and HV recanalization groups (HR: 1.02; 95% CI: 0.98–1.06, *P* = 0.29, Fig. 3d). This endpoint was not subject to significant heterogeneity (I^2^ = 44%), nor was there publication bias (supplement Fig. 1l).

## Discussion

The present meta-analysis was designed to explore immediate and long-term efficacy outcomes in BCS patients undergoing AHV recanalization. The pooled rate of clinical success rate was 96%, and these success rates were similar to those observed for patients undergoing HV recanalization. As such, the AHV can be used as an alternative to the HV to facilitate hepatic drainage in individuals diagnosed with BCS. As the AHV is not the primary mediator of hepatic drainage in healthy individuals it is often overlooked, but the hepatic hypertension that develops in individuals with BCS can result in collateral intrahepatic circulation and resultant AHV dilation [25, 26].

Restenosis is an important complication that can develop in BCS patients following recanalization, with reported restenosis rates as high as 38.3% [22]. Here the pooled AHV restenosis rate was just 17%, with this value being significantly lower than that observed following HV recanalization. The duration of primary patency was also significantly longer in the AHV group as compared to the HV group. These findings may be the result of differences in the physiology and types of obstructions associated with the AHV and HV [19]. BCS is often thought to result from HV thrombosis [27, 28]. While compensatory AHV dilation is frequently observed in individuals with BCS [10], the AHV can also ultimately be obstructed as a result of the IVC wall-mediated restriction of the AHV ostium such that this structure fails to dilate [19]. The distinct physiology underlying obstruction of the AHV and HV often results in segmental obstruction of the HV as compared to the ostial obstruction of the AHV [19].

The respective pooled primary 1- and 5-year AHV patency rates in this study were 91% and 75%, consistent with the good short-term AHV recanalization outcomes. As the 5-year AHV secondary patency rate was 91%, AHV recanalization can also be repeated when necessary.

The pooled 5-year OS rate following AHV recanalization was 95%, and no differences in OS were observed when comparing AHV and HV recanalization procedures. These findings demonstrated the lack of any significant survival benefits associated with AHV recanalization. Primary causes of mortality in individuals suffering from BCS tend to be liver failure and gastrointestinal hemorrhage following restenosis [29]. TIPS- or recanalization-based therapeutic interventions following restenosis must be performed in a timely fashion to ensure an optimal patient prognosis.

There are some limitations to this study. For one, only retrospective analyses were included. Second, these studies exhibited highly variable follow-up durations ranging from 13.7 – 60.2 months, potentially contributing to bias when evaluating long-term outcomes. Just four of the studies directly compared outcomes between HV and AHV recanalization procedures, and while no significant heterogeneity pertaining to the analyzed endpoints was detected, the statistical power available for these comparisons was nonetheless limited. Moreover, one of these studies [20] only reported on AHV stent insertion in BCS patients. Stenting is generally formed when balloon dilation procedures are a technical failure, and these AHV stenting results may thus not accurately reflect the true clinical efficacy of AHV recanalization. Thirdly, compensatory AHVs are not present in all BCS patients, thus inherently restricting the viability of this recanalization approach to a limited patient subset. Finally, all included studies are from China. Therefore, these pooled results may lack the representiveness from all of the world.

## Conclusion

In summary, the present results highlight the promise of AHV recanalization as an effective means of treating BCS patients that is associated with a satisfactory long-term prognosis, potentially resulting in better long-term outcomes than those associated with HV recanalization.

### Supplementary Information

**Additional file 1: Supplement Fig. 1.** The funnel plots of the endpoints of (a) clinical success rate, (b) AHV pressure before and after recanalization, (c) 1-year primary patency rate, (d) 5-year primary patency rate, (e) 1-year secondary patency rate, (f) 5-year secondary patency rate, (g) 1-year OS rate, (h) 5-year OS rate, (i) comparative clinical success rates, (j) comparative re-stenosis rates, (k) primary patency duration, and (l) OS duration.

**Additional file 2. **

## Data Availability

The data that support the findings of this study are available from the corresponding author upon reasonable request.

## Abbreviations

AHV: Accessory hepatic vein
BCS: Budd-Chiari syndrome
HV: Hepatic vein
OS: Overall survival
TIPS: Transjugular intrahepatic portosystemic shunt
