# Supplementary figures and images for: Accessory hepatic vein recanalization for Budd-Chiari syndrome: a systematic review and meta-analysis
Source: BMC Gastroenterol. 2023 Oct 2;23:340. doi: 10.1186/s12876-023-02969-z (PMC10546748; doi:10.1186/s12876-023-02969-z)

a
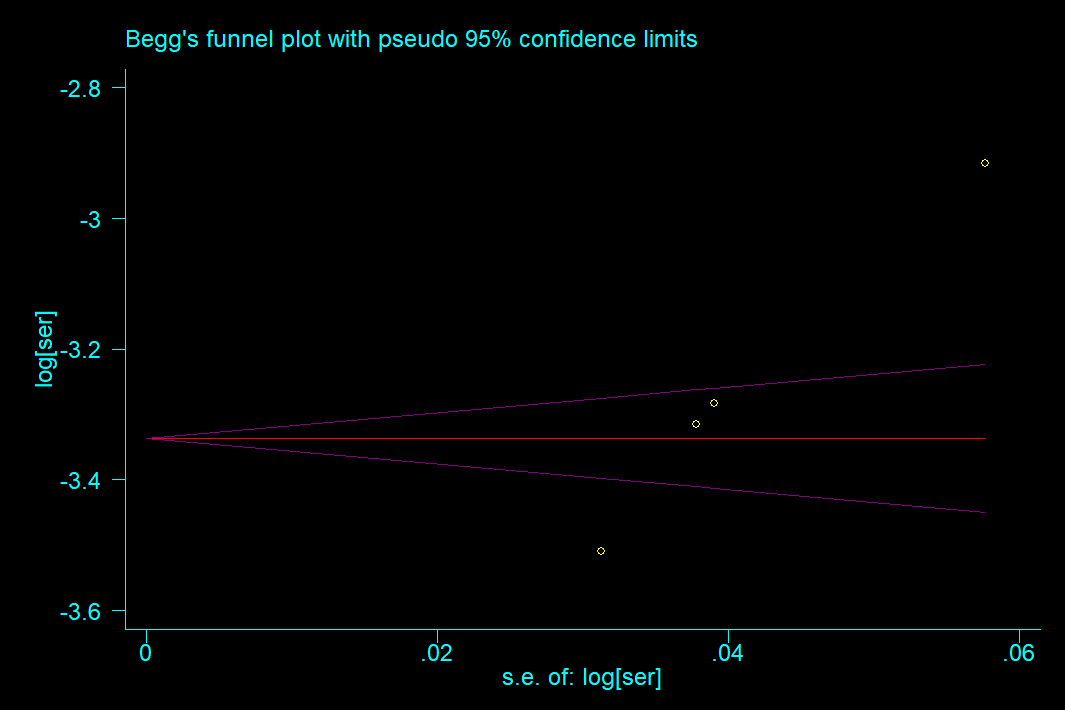


b
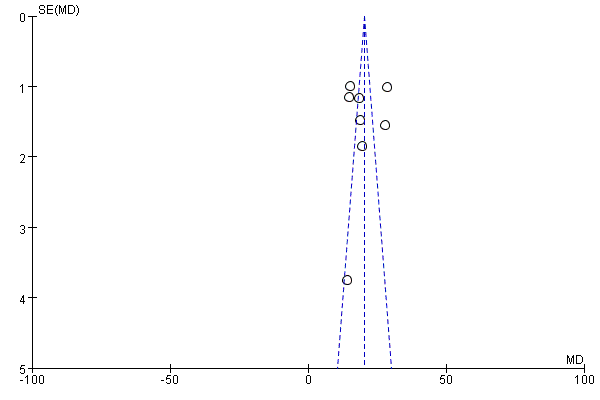


c
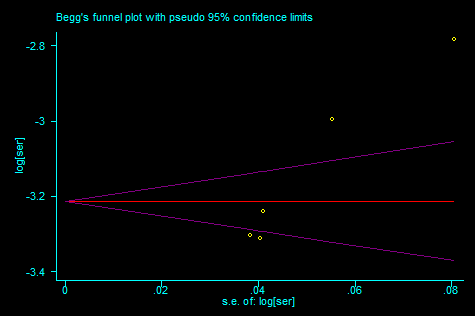


d
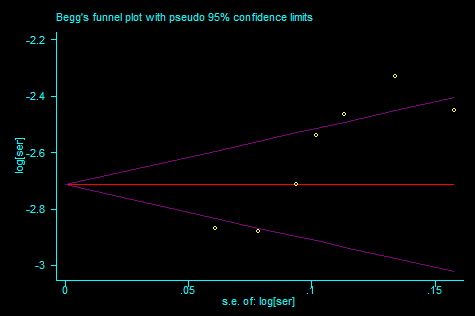


e
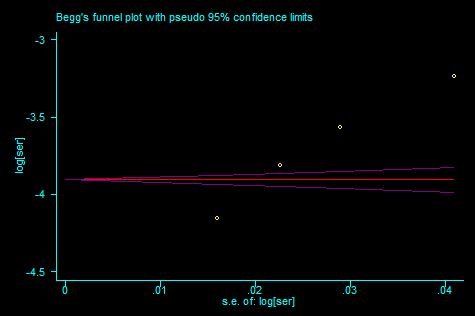


f
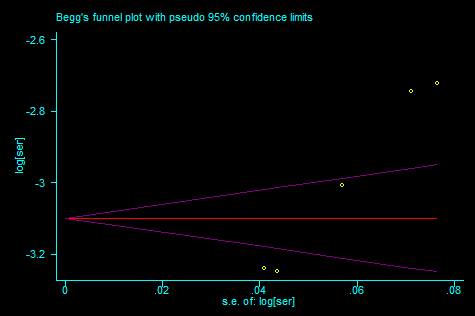


g
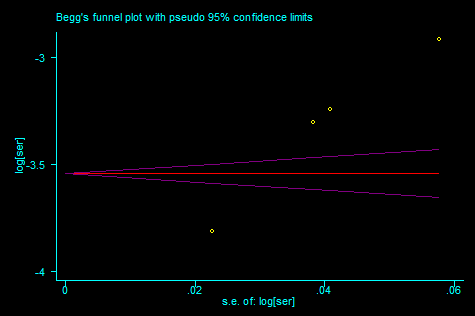


h
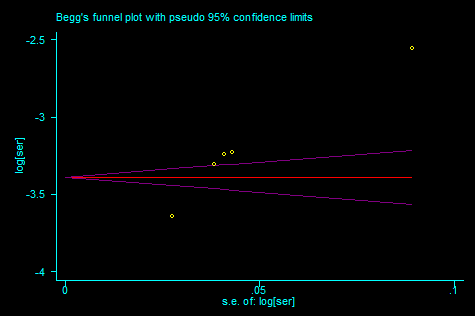


i
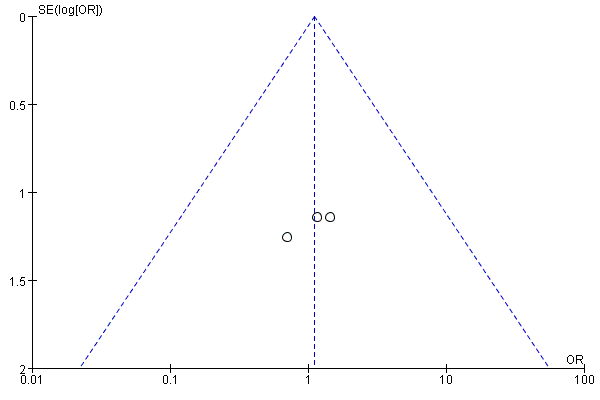


j
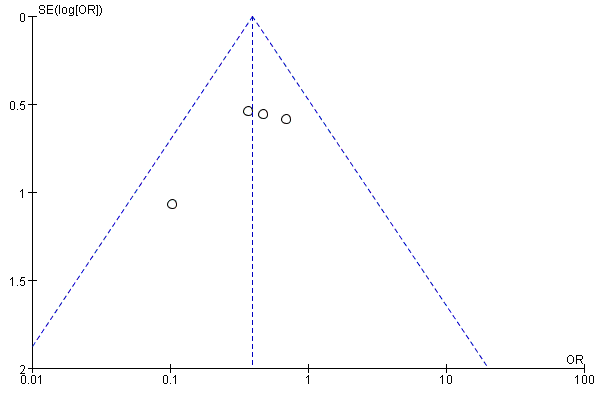


k
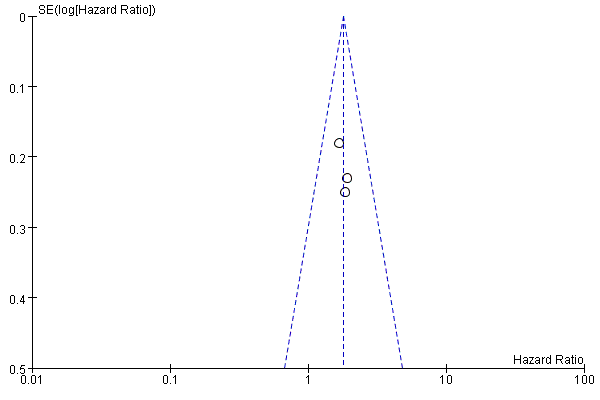


l
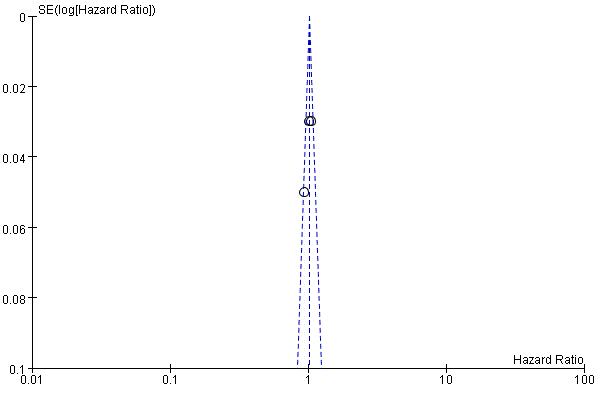

Supplement: Supplementary file 1 — Additional file 1: Supplement Fig. 1. The funnel plots of the endpoints of (a) clinical success rate, (b) AHV pressure before and after recanalization, (c) 1-year primary patency rate, (d) 5-year primary patency rate, (e) 1-year secondary patency rate, (f) 5-year secondary patency rate, (g) 1-year OS rate, (h) 5-year OS rate, (i) comparative clinical success rates, (j) comparative re-stenosis rates, (k) primary patency duration, and (l) OS duration. [file 12876_2023_2969_MOESM1_ESM.doc]
